# Case Report: Hereditary Alpha Tryptasemia in Children: A Pediatric Case Series and a Brief Overview of Literature

**DOI:** 10.3389/fped.2021.716786

**Published:** 2021-08-20

**Authors:** Daniele Zama, Edoardo Muratore, Arianna Giannetti, Iria Neri, Francesca Conti, Pamela Magini, Simona Ferrari, Andrea Pession

**Affiliations:** ^1^Pediatric Unit, IRCCS Azienda Ospedaliero-Universitaria di Bologna, Bologna, Italy; ^2^Dermatology Unit, IRCCS Azienda Ospedaliero-Universitaria di Bologna, Bologna, Italy; ^3^Unità Genetica Medica, IRCCS Azienda Ospedaliero-Universitaria di Bologna, Bologna, Italy

**Keywords:** hereditary alpha tryptasemia, mastocytosis, mast cell activation syndrome, pediatric allergology, genetic disorder

## Abstract

Hereditary alpha tryptasemia (HαT) is a recently described autosomal dominant genetic trait caused by an increased copy number of the TPSAB1 gene. It commonly leads to elevated basal serum tryptase levels, and it is associated with heterogeneous clinical manifestations. Some individuals report few to no symptoms, while others present with a spectrum of debilitating features. Most symptoms related to HαT may be explained by mast cell activation and mediator release, namely multiple allergies, anaphylaxis, and skin rash. However, the genotype-phenotype correlation has not yet been clearly understood. In particular, the characterization of the clinical spectrum lacks in children, where differential diagnosis could be challenging. Systemic mastocytosis, HαT, and mast cell activation syndrome are all associated with overlapping pathophysiology and symptoms, making the distinction between these conditions a difficult task. We herein describe two pediatric cases of HαT and their respective families at our tertiary care teaching hospital, highlighting the diagnostic workup and differential diagnosis. We also provide a brief review of the literature to underline the peculiar features of this condition in children.

## Introduction

Hereditary alpha tryptasemia (HαT) is a highly penetrant autosomal dominant genetic trait associated with an increased copy number of the TPSAB1 gene on chromosome 16 (1). The alpha-tryptase-encoding locus presents a high frequency of copy number variations. For example, 29% of people express no alpha-tryptase alleles without clinical manifestations, more commonly in Caucasians (up to 45%) and less frequently in other ethnical background (2). In addition to the canonical genotypes, increased germline TPSAB1 copy number has recently been described in association with elevated basal serum tryptase and clinical features compatible with mast cell activation syndrome (MCAS) (1).

This new genetic condition, HαT, described by Lyons et al. in 2016, potentially explains the majority of cases with slightly elevated basal serum tryptase (3, 4). The upper limit of normal serum tryptase is mainly defined as 11.4 ng/ml, and it is believed to be elevated at baseline in a high percentage of Caucasians, around 5% (5). Most patients with increased TPSAB1 copy number have elevated basal serum tryptase levels (1, 6, 7). The clinical expressivity of the genetic trait is variable and not yet fully characterized. Some individuals report few to no symptoms, while others present a spectrum of debilitating clinical features. Recurrent cutaneous symptoms, like urticaria, angioedema, and skin flushing, are commonly reported (1, 4). Gastrointestinal complaints are also frequent, comprising abdominal pain, gastroesophageal reflux, diarrhea, and food intolerances, sometimes meeting the required criteria for the diagnosis of functional gastrointestinal disorders (1, 4). Systemic allergic reactions and anaphylaxis are key clinical features, most notably triggered by hymenoptera venom (8). Autonomic dysfunction, comprising postural tachycardia syndrome and hypotension, retained primary dentition, behavioral complaints, sleep disturbance, chronic pain, and joint hypermobility, are also reported (1, 4, 6). Moreover, a higher prevalence of increased TPSAB1 germline copy number was observed in a cohort of patients with mastocytosis than the general population (17.2 vs. 4.4%), suggesting a possible correlation between the two conditions (9) (Table 1).

**Table 1:** Summary of studies regarding HαT.

**First author**	**Year**	**Reference**	**Number of HαT patients**	**Population**	**Copy number variations in the locus**	**Basal serum tryptase**	**Clinical characteristics**	**Other results**
Lyons	2016	(1)	35 families, 96 cases	Mean age 39 years, range 2–89	73 duplications 15 triplications	Median 15.9 ng/ml, interquartile range 12.6–20.7 (Duplications 14.3 vs. Triplications 23.4)	Systemic venom reaction 16%; Flushing/pruritus 51%; Irritable bowel syndrome 49%; Chronic gastroesophageal reflux symptoms 65%; Congenital skeletal abnormality 26%; Retained primary dentition 21%; Hypermobility 28%; Autonomic syndrome 47%; Positive tilt-table test 11%; Arthralgia 45%; Body pain/headache 47%; Sleep disruption 39%	Findings confirmed in two independent cohorts (172 patients, of which 8 and 9 with HαT) Individuals harboring alleles encoding three copies of α-tryptase had higher basal serum levels of tryptase and were more symptomatic
Sabato	2018	(20)	1 family, 7 cases	5 adults, 2 children	Quintuplication	Median 35 ng/ml	Four were symptomatics, with recurrent episodic of severe abdominal pain and diarrhea. One of them was a 22-year-old male with also hepatosplenomegaly and a somatic cKIT mutation	–
Lyons	2018	(6, 21)	46 families, 57 cases	Between the ages of 45 and 65, with and without CACNA1H variant	Duplications	Median 15.6 ng/ml in patients with CACNA1H variant vs. 13.5 ng/ml in wild type	The variant CACNA1H haplotype does not result in detectable phenotypic differences in the heterozygous state	32/46 families were found to have the haplotype containing three functional CACNA1H variants, which *in vitro* imparts partial gain of function
Robey	2020	(4)	70 cases	Median 41 years, range 3–88. 24% under 18 years old	79% with a duplication; 21% with duplications of both alleles, triplications or quintuplications	Median 17.0 ng/ml, range 7. 6–60.9 (Duplications 15.2 vs. higher copy number 22.0)	Urticaria/angioedema 51%; skin flushing 41%; abdominal pain;43% loose stool 36%; aches and pains 41%; joint hypermobility 17%; behavioral changes/“brain fog” 24%; Postural Tachycardia Syndrome 16%	Clinical manifestations were not more common in patients with higher alpha-gene copy number than in those with duplications. α-tryptase duplication was present in 5% of an unselected British birth cohort
Lyons	2020	(8)	91 cases	Patients with venom anaphylaxis, idiopathic anaphylaxis, systemic mastocytosis, healthy individuals, and control with non-atopic disease	3 triplications, 25 duplications in healthy individuals and control with non-atopic disease 4 duplications, 5 triplications, 1 quadruplications in systemic mastocytosis Not reported in venom and idiopatic anaphylaxis	–	HαT was associated with grade IV venom anaphylaxis, mainly in individuals with idiopathic anaphylaxis and systemic mastocytosis. Increased risk of anaphylaxis in patients with mastocytosis.	Protease-activated receptor-2-dependent vascular permeability was induced by tryptase heterotetramers but non-homotetramers
Carrigan	2020	(3)	2 cases	1 adult and 1 child	Duplications	From 11.9 to 13.4 ng/ml in the child and from 13.0 to 16.0 ng/ml in the adult	Child: recurrent flushing which improved with antihistamines. Abdominal pain with loose stools. Numerous foods caused flushing, bloating, constipation, and abdominal pain Adult: history of syncopal and presyncopal episodes accompanied by itchy skin, facial flushing, and swelling. Dysautonomia with a positive tilt-table test. Bloating, nausea, vomiting, abdominal cramping, and loose stools. Hymenoptera sting caused generalized urticaria, wheezing, chest pain, and life-treating anaphylaxis	Potential utility and limitations of genetic HαT testing while evaluating patients with symptoms of mast cell activation and elevated basal serum tryptase
Greiner	2021	(9)	85 cases	Mastocytosis discovery cohort: median age 48 year (range 11–91) Mastocytosis validation cohort: median age 49 years (range 23–71) Other myeloid neoplasm cohort: median age 59 years (range 12–89)	33 duplications, 46 triplications, 6 quadruplications or more	Patients with mastocytosis and HαT exhibited higher tryptase levels than patients without HαT independent of mast cell burden (median tryptase 49.6 vs. 34.5 ng/ml)	Hymenoptera venom hypersensitivity reactions and severe anaphylaxis were more frequently observed in mastocytosis patients with HαT than in those without HαT	HαT was identified in 17.2% of mastocytosis patients and 4.4% of the control population, hinting at a potential pathogenic role

The characterization of the clinical spectrum of this condition lacks in children, where differential diagnosis could be challenging. We herein describe two pediatric cases of HαT and their respective families that occurred at our tertiary care teaching hospital, in the pediatric unit of the “IRCCS Azienda Ospedaliero-Universitaria di Bologna.”

## Case Presentation

### Case 1

**Figure 1:**
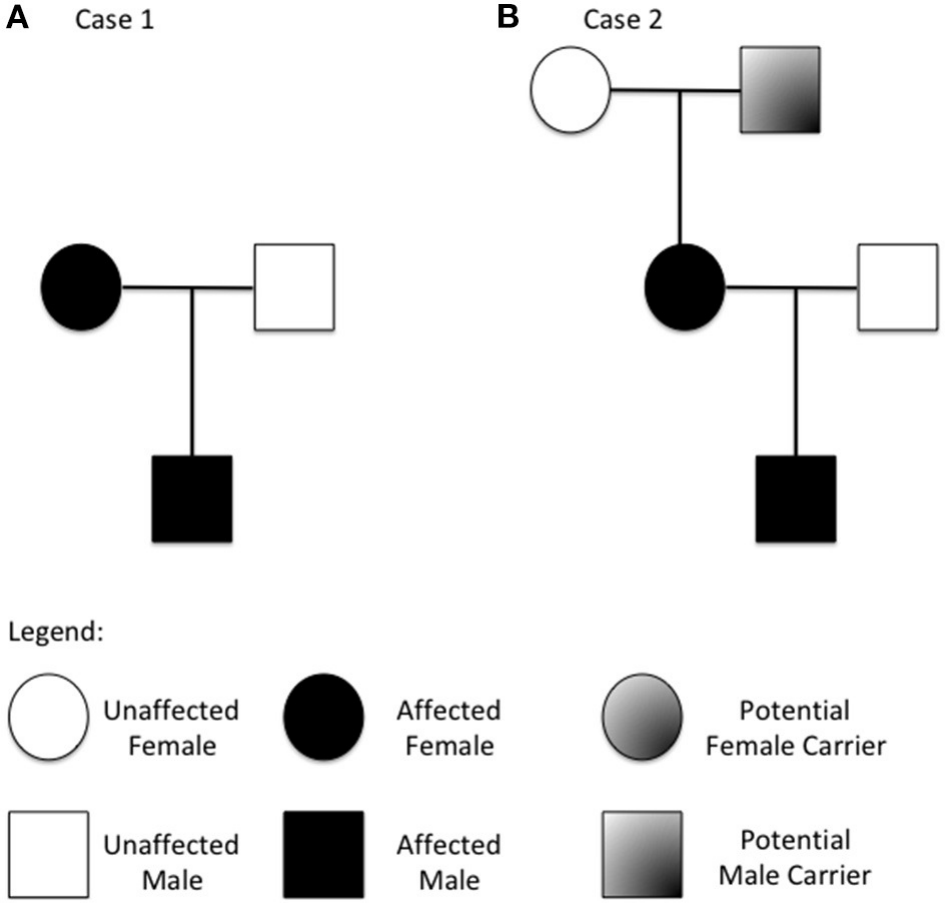
Family tree from the two described families (**A** refers to case 1 and **B** to case 2). Patients with increased copy number of the *TPSAB1* gene are highlighted.

We describe a two-generation family of Russian origins. The proband is a 4-year old boy referred to our center for suspect systemic mastocytosis (Figure 1A).

The proband's mother is a 28 years old woman, previously diagnosed with allergic asthma, Hashimoto thyroiditis, and type 1 diabetes mellitus complicated by necrobiosis lipoidica. She also had numerous allergic reactions to drugs and food in addition to multiple episodes of anaphylaxis after hymenoptera stings and others not specified triggers. The patient also experienced chronic diarrhea (2–20 episodes per day), autonomic dysfunction (recurrent syncope and hyperhidrosis), recurrent papular skin lesions and flushing, juvenile osteoporosis with T8 fracture, multiple lymphadenomegaly, and multiple miscarriage in the first trimester of pregnancy. The diagnostic work-up revealed increased levels of bee venom-specific IgE and elevated basal serum tryptase.

A bone marrow biopsy was performed to exclude systemic mastocytosis that revealed no morphologic, immunophenotyping, and cytogenetic abnormalities. The research of c-KIT D816V mutation was negative. Esophagogastroduodenoscopy and colonoscopy with multiple biopsies were also negative. Thus, a diagnosis of MCAS was made. She was initially treated with intravenous corticosteroids and interferon, and she is currently receiving oral antihistaminic before meals and pancreatic enzymes. The father of the proband reported no relevant clinical history.

The proband was born at 31 weeks of gestational age by cesarean section due to maternal pathology. The pregnancy was complicated by intrauterine growth restriction and agenesis of the ductus venosus. At birth, the patient weighed 1,838 g and had moderate respiratory distress, jaundice, hypoglycemia, transitory elevated TSH due to transplacental passage of maternal autoantibody and anemia, and was therefore transferred to neonatal intensive care unit for 1 month. During the first months of life, he presented repeated episodes of vomiting (2–3 episodes/day) without relation with the meal, for which a diagnosis of gastroesophageal reflux was made. Starting from about 1 year of age, he experienced multiple episodes of acute otitis media and tonsillitis treated with antibiotics, and daily episodes of bronchospasm treated initially with inhaled short-acting beta2 agonist and cortisone and afterward with inhaled corticosteroid only. From 15 months of age, he started to present multiple daily episodes of diarrhea (3–11 episodes per day) and abdominal pain exacerbated by meals. Celiac disease screening, stool culture, total Ig, and IgE levels, skin prick test for inhalant allergens, and RAST for alimentary allergens were all negative, whereas an elevated basal serum tryptase was observed (13 ng/ml). Symptoms worsened in the summer and partially responded to a clinical trial of cetirizine and nedocromil but recurred after therapy withdrawal.

Due to recurrent upper airway infection, the patients underwent rhinolaryngoscopy, which revealed adenoid hypertrophy. The proband also presented with different episodes of skin rash associated with hot bath, administration of ibuprofen, and upper airway infection. These symptoms receded after the administration of ketotifen. Moreover, the family reported significant local reactions to insect stings. An increment in the level of basal serum tryptase compared to the previous control was also observed (17 ng/ml). Clinical and laboratory findings, taken together with family history, raised the suspicion of systemic mastocytosis, and therefore the proband was referred to our center at 2 years of age.

At our referral center, the child underwent a complete diagnostic work-up. Basal serum tryptase was found to be 16.4 ng/ml. Levels of Vitamine D 25 OH were reduced (12 ng/ml, n.v. > 20 ng/ml), with slight elevation in alkaline phosphatase and osteocalcin levels and normal parathormone. Fecal calprotectin was also increased (405 mcg/g, n.v. <50 mcg/g). Complete blood count, liver and kidney function total serum Ig, Ig subclasses, and lymphocyte subset were normal, as well as complete abdominal ultrasound.

In the suspicion of systemic mastocytosis, we performed bone marrow biopsy, esophagogastroduodenoscopy, and colonoscopy with multiple biopsies, finding no evidence of mast-cell infiltration. The analysis of the c-KIT D816V mutation was negative on bone marrow specimen. Systemic mastocytosis was therefore excluded, and a diagnosis of MCAS was proposed.

Clinical re-evaluation in the following period highlighted red dermographism, iron deficiency, reduced attention and hyperactivity, recurrent skin rash, and diarrhea generally exacerbated by hot temperatures (e.g., in the summer and after exercise). Multiple episodes of anaphylaxis occurred after hymenoptera stings and others not clearly specified triggers. Moreover, the patient experienced laryngeal edema during laryngitis that required multiple intramuscular adrenaline injections and prompted hospitalization. In consideration of these multiple life-threatening events, an adrenaline autoinjector was prescribed to be held at home.

Basal serum tryptase levels slightly decreased over time, with the highest value being 14.7 ng/ml and the lowest 11 ng/ml. The gastrointestinal complaints were treated first with cromoglycate and then with ketotifen, but in both cases, treatment was suspended due to constipation.

In the suspicion of HαT, genetic testing was performed on the proband and parents. qPCR assay was done with the Universal ProbeLibrary System (Roche). A specific region within the TPSAB1 gene was amplified using the UPL probe 38 and the following flanking primers: forward 5′-aacccctgctgtccaaaac-3′; reverse 5′-taatgaggtccagcactcagg-3′ (chr16:1,292,280–1,292,539, hg19). Copy number variation was calculated with the ΔΔCt method, using HTR7 as the reference gene for normalization and two DNA samples from healthy donors as control. Duplication of the TPSAB1 gene was found in the proband and the mother but not in the father.

### Case 2

Herein, we describe a three-generation family of Italian origins. The proband is a 2 years old boy referred to our center for suspected Food-Protein induced Enterocolitis (Figure 1B).

The mother of the proband is a 31 years old woman. She reported allergic rhinoconjunctivitis and recurrent gastrointestinal complaints from a young age, described as frequent loose stools and abdominal pain, interpreted as food allergies. She also experienced dizzy spells, vertigo, and small skin rash. Laboratory findings included normal Ig levels, normal total IgE levels, and no specific Ig for known allergens, whereas increased basal serum tryptase was found. The maternal grandfather also reported non-specific gastrointestinal symptoms and elevated serum tryptase. The father of the proband had no relevant clinical history.

**Figure 2:**
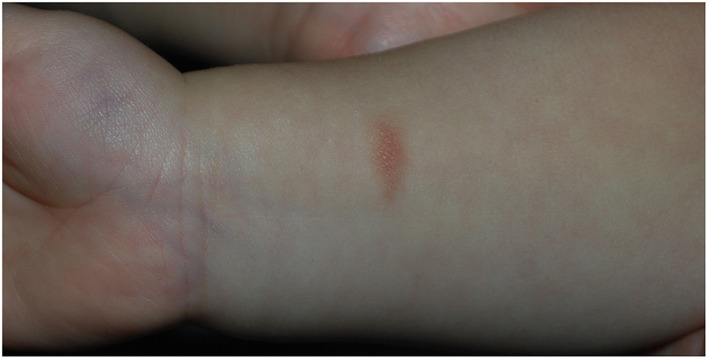
Solitary cutaneous mastocytoma of case 2, presenting as a maculo-papular hypercromic lesion with positive Darier sign in the right wrist.

The proband was born at term via vaginal delivery from an uncomplicated pregnancy. The baby at birth weighed 2,750 g. He received mixed feeding for the first month and only breastfeeding in the following period. He then developed cradle cap and peri-auricular dermatitis. Gastroesophageal reflux was also diagnosed. The child started weaning at 6 months of age, and after the introduction of wheat at 8 months, he developed facial erythematous skin rash and diarrhea (5–6 episodes per day) starting 2 h after the aliment ingestion. After wheat reintroduction, the symptoms recurred, and so it was excluded from the diet. He then also experienced episodes of diarrhea after 3–4 h from the intake of milk derivatives and vomiting linked to the ingestion of egg yolk, which led to the exclusion of these two aliments. Skin prick test for alimentary allergens resulted negative. In the following weeks, reintroducing small quantities of wheat was attempted, but that prompted a severe episode of vomiting that required hospitalization. A diagnosis of Food-Protein induced Enterocolitis was therefore suspected. At 1 year of age, he presented atopic dermatitis and a maculopapular hyperchromic lesion with a positive Darier sign in the right wrist, compatible with solitary cutaneous mastocytoma (Figure 2). Basal serum tryptase was 19.8 ng/ml. Levels of Vitamine D 25 OH, alkaline phosphatase, and parathormone were normal. Total IgE levels were also normal, but positive specific IgE antibodies for dust-mite were found in the absence of related respiratory symptoms. After the first trial of cow-milk reintroduction, the proband experienced upward and rightward gaze deviation followed by generalized hypotonia and lack of responsiveness lasting about 5 s, with normal vital signs. Brief episodes of gaze deviation recurred weekly in the following months, associated with forward movements of the neck and reduced responsiveness. They mainly happened when the child was tired or agitated. No other neurological alteration was found.

The patient presented also sleep disturbances, including insomnia, pavor nocturnus, and somnambulism. Polysomnography ruled out the epileptic origin of these signs. Basal serum tryptase levels fluctuated in the following months, from 15.8 to 18.8 ng/ml. In order to better characterize the reason for the persistent rise in serum tryptase, the proband, the mother, and the father underwent genetic testing on the TPSAB1 gene, using the same method as previously described in Case 1. Triplication (four copies) of the TPSAB1 gene was found in the proband and the mother but not in the father.

After genetic testing, progressive reintroduction of food was attempted. Cow milk, wheat, and egg were successfully reintroduced in the diet without additional specific treatments. Neurological symptoms also receded with time.

## Discussion

Hereditary alpha tryptasemia is a recently described, apparently common genetic trait affecting mast cells (MC). Herein, we described four cases from two different families focusing on the two pediatric patients, characterized by remarkably different clinical presentation and severity. The genotype–phenotype correlation in this genetic condition still needs to be clearly understood (1) and, in particular, how increased copy number of the TPSAB1 gene could lead to elevated basal serum tryptase level and the associated heterogeneous clinical manifestations.

The multiple tryptase genetic loci in humans are located within the gene-rich region at 16p13.3 and include four tryptase encoding genes (TPSG1, TPSB2, TPSAB1, and TPSD1), of which only two, TPSB2 and TPSAB1, encode the secreted isoforms. The TPSAB1 locus encodes either alpha or beta isoforms, while TPSB2 is considered to encode only beta tryptase (6, 10). These isoforms are 97% similar, making detection of distinct types extremely difficult (6).

Tryptases are trypsin-like serine proteases expressed by MC and basophils (11, 12). The biologic activity of the enzymatically active tetrameric form has not yet been fully understood, but it is speculated to contribute to allergic inflammation (13). Alpha-tryptase tetramers lack protease activity, whereas β-tryptase tetramers are active proteases. Heterotetramers composed of 2α- and 2β-tryptase, but not homotetramer, activate protease-activated receptor-2 (PAR2), expressed on smooth muscle, neurons, and endothelium. Also, only α/β-tryptase tetramers cleave the α subunit of the EGF-like module-containing mucin-like hormone receptor-like 2 (EMR2) mechanosensory receptor, making MC susceptible to vibration-triggered degranulation. The allosteric effect of alpha tryptase is supposed to explain the different biological effects of heterotetramers compared to homotetramers (14). Pro-tryptases, which have not undergone enzymatic conversion into mature tetrameric tryptases, are constitutively secreted into serum in their monomeric form by MC at rest, and provide the vast majority of measured basal serum tryptase in a healthy individual. In contrast, mature tryptases are stored in secretory granules until their release by activated cells (15). Mast cell release tetrameric tryptases during a severe anaphylactic event, and serum tryptase level increases substantially over the individual's baseline. Therefore, serum tryptase is a valuable biomarker for assessing MC burden (as in mastocytosis) and, in some settings, MC activation (16). Increased copy number of the TPSAB1 gene could thus directly explain the increased basal serum tryptase levels observed in HαT patients. Other diseases in which elevated total tryptase levels could be found are acute myeloid leukemia, myelodysplastic syndromes, hypereosinophilic syndrome with the FLP1L1-PDGFRA fusion gene, and end-stage renal disease (16, 17).

Most symptoms related to HαT may be explained by MC mediator release, namely multiple allergies, anaphylaxis, and skin rash (6). Moreover, the portion α/β-tryptase heterotetramers increase with the alpha-tryptase gene dosage, and this could explain the connective tissue abnormalities, such as joint hypermobility, through the activation of PAR2-dependent pathways. The stimulation of the same receptor in the endothelium could also potentiate hypotension and anaphylaxis by increasing vascular permeability (18). Some cases of vibratory urticaria or other allergic manifestations are, instead, probably associated with higher susceptibility of MC to vibration-triggered degranulation due to the increased cleavage of EMR2α (14). Hereditary alpha tryptasemia is also associated with increased small intestinal MC, located throughout the mucosa and submucosa, including the superficial villi, contributing to the observed gastrointestinal manifestations (19).

No generalized conclusions or statements can be derived from the description of our two families. Moreover, the presented cases are influenced by selection bias, given that their tryptase levels would not have been checked if they did not present with their clinical manifestations. Larger case series are needed to understand whether there is a pattern or a clinical effect of HaT in the pediatric population. Moreover, HαT may modify the expression of multifactorial allergic diseases rather than directly cause specific phenotypes (4). Patients with mastocytosis, venom allergies, or idiopathic anaphylaxis have more severe manifestations of anaphylaxis if they also present alpha tryptase encoding TPSAB1 copy number gain (8, 9), possibly indicating that HaT could be a disease-modifying factor rather than a disease-causing mutation. Further studies are needed to address the specific role of HαT in the genesis of the associated symptoms.

Interestingly, in our experience, the first patient had a more severe clinical course, but with lower levels of basal serum tryptase and fewer *TPSAB1* gene copies, whereas the second showed a milder clinical course, although he had *TPSAB1* triplication and higher levels of tryptase. The described cases seem to contrast with the hypothesis of a linear gene-dosage effect between the number of additional *TPSAB1* copies, the basal serum tryptase level, and the severity of clinical symptoms (20). The presented cases are instead in line with the idea that no precise genotype–phenotype correlation could be established between TPSAB1 copy number variation and clinical presentation (4). Thus, other genetic modifiers may contribute to shaping the clinical phenotype. Due to the genetic complexity of the tryptase locus, the germline sequence of this portion of the genome has not been entirely determined. Therefore, coinherited variants may contribute to one or more heterogeneous clinical manifestations observed among individuals affected by HαT. To date, no such variants were linked to a specific phenotype (21).

The first patient eventually received a diagnosis of MCAS after the exclusion of systemic mastocytosis. Mast cell activation syndrome is defined as excessive MC activity without clonal proliferation or obvious secondary triggers (7). The key feature for the diagnosis is recurrent episodes of systemic anaphylaxis with concurrent involvement of at least two among cardiovascular, respiratory, dermatologic, and gastrointestinal systems. The clinical symptoms have to be associated with an acute increase in specific biologic mediator levels secreted by MC (e.g., histamine, tryptase, prostaglandin D2, Leukotriene C4) (22). Clinical heterogeneity in MCAS patients likely is due to MC mutational complexity and heterogeneity. Few studies have investigated the genetic basis of MCAS, reporting associations with somatic mutations in KIT and other MC regulatory genes, but with the possibility of germline mutations occurring (23).

The second proband also presented a solitary cutaneous mastocytoma. A higher prevalence of HαT in patients with mastocytosis compared to matched-control subjects has been recently reported, even if with lower frequency in cutaneous mastocytosis than systemic forms (9). The pathogenic role of germline α-tryptase encoding TPSAB1 copy number gains in the development of clonal MC infiltration of the skin or other organs still needs to be understood. It is speculated that increased α-tryptase load could exert direct mitogenic activity on MC or influence the microenvironment with autocrine or paracrine mechanism, facilitating clonal proliferation (24–26).

Mastocytosis is defined by clonal MC expansion and accumulation in various tissues, mainly the bone marrow and skin (27). Clinical presentations reflect organ damage due to MC infiltration and symptoms of MC activation, similar to MCAS (11). The diagnosis of systemic mastocytosis is based on the criteria established by the WHO (28). Total serum tryptase levels should be measured in all patients with symptoms compatible with mastocytosis at least 24 h after the symptomatic event. If the basal tryptase level is ≥20.0 ng/ml, the likelihood of systemic mastocytosis is high, and a bone marrow biopsy is warranted. Normal or slightly elevated basal serum tryptase levels (11.5–20.0 ng/ml) do not rule out the diagnosis, and highly sensitive polymerase-chain-reaction techniques for KIT D816V transcripts in peripheral-blood leukocytes could be used to confirm or exclude the clinical suspicion (29). For patients in whom the KIT mutation is detected in peripheral blood, a bone marrow evaluation is indicated (11). Due to high clinical suspicion, bone marrow and gastrointestinal biopsies were performed directly in the first patient, even with levels of tryptase <20.0 ng/ml. The exclusion of MC clonal proliferation in the bone marrow and the gut prompted the diagnosis of MCAS.

The most common form of mastocytosis in children is cutaneous mastocytosis. Disease-onset is usually within the first 2 years of life and commonly experience spontaneous regression of skin lesions at puberty (7). Three major variants are defined: monomorphic and polymorphic maculopapular cutaneous mastocytosis, diffuse cutaneous mastocytosis, and solitary mastocytoma of skin (30). The hallmark of cutaneous mastocytosis is Darier's sign, which is the lesions that become urticarial when rubbed or scratched (11). Children with typical cutaneous lesions usually do not require a bone marrow biopsy if hepatosplenomegaly, lymphadenopathy, or peripheral-blood abnormalities are absent (11). Patients with cutaneous mastocytosis do not fulfill the criteria for the diagnosis of systemic mastocytosis. However, these patients could also present systemic symptoms such as anaphylaxis or gastrointestinal complaints, similar to those documented in MCAS. In this regard, the second proband presented both a solitary mastocytoma of the skin and Food-Protein induced Enterocolitis, but bone marrow examination was not performed because no indication of systemic MC proliferation was found. Nevertheless, elevation in basal serum tryptase levels prompted the genetic testing on the TPSAB1 gene.

The recent association of increased *TPSAB1* copy number with HαT, with few supporting scientific publications and the complex structure of the genomic region, likely contribute to a delay of the diagnostic offer by public institutions. Since the increased copy number cannot be easily identified through routine genetic testing, no public laboratories routinely offer this test in Europe. A commercial one is offered by at least one company in the USA (Gene by Gene, Houston, Texas). However, specialists, including medical geneticists, should evaluate the access to a targeted genetic test for TPSAB1 copy number and its results. Moreover, the clinician must consider the differential diagnostic possibility of other genetic diseases, such as congenital autoinflammatory syndromes or congenital malabsorption syndromes, mainly inborn disaccharidase deficiency, that were excluded in our patients.

Systemic mastocytosis, HαT, and MCAS are associated with overlapping symptoms and pathophysiology, and distinguishing them only on clinical grounds could be challenging (3). Pediatric patients with increased basal serum tryptase should be all screened for increased copy number variations in the TPSAB1 locus, the genetic hallmark of HαT. If compatible symptoms and slightly elevated tryptase levels are present, HαT could be diagnosed, and further diagnostic testing is not needed. However, if clinical and laboratory findings suggest mastocytosis, the diagnosis of HαT must not rule out the need for an invasive investigation because the two conditions could coexist. More extensive studies are therefore needed to establish the role of tryptase genotyping in the diagnostic algorithm when considering the necessity for bone marrow biopsy.

An inadequate diagnostic pathway in children could cause late diagnosis of these conditions, leading to a reduction in the quality of life and several unnecessary medical consultations and therapies, as highlighted by the description of our families. Clinical overlap with common diseases, such as functional gastrointestinal disorders and allergies, makes the diagnosis of HαT in adulthood a problematic task. Therefore, suspecting this condition in children based on clinical phenotype and family history should prompt genetic testing to perform early diagnosis.

The observed heterogeneity in HαT clinical presentation requires further studies to better characterize clinical phenotype in children. The exact correlation between clinical findings and genetic alteration is unclear and should be a research focus in the next future. A better understanding of the associated symptoms could improve diagnosis and genetic counseling, helping physicians manage patients with MCAS-related symptoms previously considered of idiopathic origin.

## Data Availability Statement

The original contributions presented in the study are included in the article/supplementary material, further inquiries can be directed to the corresponding author/s.

## Ethics Statement

Ethical review and approval was not required for the study on human participants in accordance with the local legislation and institutional requirements. Written informed consent to participate in this study was provided by the participants' legal guardian/next of kin. Written informed consent was obtained from the minor(s)' legal guardian/next of kin for the publication of any potentially identifiable images or data included in this article.

## Author Contributions

DZ and EM: conceptualization. AG, IN, SF, PM, and EM: data collection. EM and DZ: writing—original draft preparation. EM: image and table design. AG, IN, FC, PM, SF, and AP: writing—review and editing. All authors have read and agreed to the published version of the manuscript.

## Conflict of Interest

The authors declare that the research was conducted in the absence of any commercial or financial relationships that could be construed as a potential conflict of interest.

## Publisher's Note

All claims expressed in this article are solely those of the authors and do not necessarily represent those of their affiliated organizations, or those of the publisher, the editors and the reviewers. Any product that may be evaluated in this article, or claim that may be made by its manufacturer, is not guaranteed or endorsed by the publisher.
